# Coexpression of epidermal growth factor receptor with related factors is associated with a poor prognosis in non-small-cell lung cancer

**DOI:** 10.1038/sj.bjc.6602149

**Published:** 2004-09-07

**Authors:** D E B Swinson, G Cox, K J O'Byrne

**Affiliations:** 1 Cancer Centre, Queen Elizabeth Hospital, Edgbaston, Birmingham B15 2TH, UK; 2 Kingsmill Hospital, Mansfield, Nottingham NG1 1AA, UK; 3 Thoracic Oncology Research Group, St James's Hospital, Dublin 8, Ireland, UK

**Keywords:** CA IX, NSCLC, EGFR, MMP-9

## Abstract

The epidermal growth factor receptor (EGFR) is commonly expressed in non-small-cell lung cancer (NSCLC) and promotes a host of mechanisms involved in tumorigenesis. However, EGFR expression does not reliably predict prognosis or response to EGFR-targeted therapies. The data from two previous studies of a series of 181 consecutive surgically resected stage I–IIIA NSCLC patients who had survived in excess of 60 days were explored. Of these patients, tissue was available for evaluation of EGFR in 179 patients, carbonic anhydrase (CA) IX in 177 patients and matrix metalloproteinase-9 (MMP-9) in 169 patients. We have previously reported an association between EGFR expression and MMP-9 expression. We have also reported that MMP-9 (*P*=0.001) and perinuclear (p)CA IX (*P*=0.03) but not EGFR expression were associated with a poor prognosis. Perinuclear CA IX expression was also associated with EGFR expression (*P*<0.001). Multivariate analysis demonstrated that coexpression of MMP-9 with EGFR conferred a worse prognosis than the expression of MMP-9 alone (*P*<0.001) and coexpression of EGFR and pCA IX conferred a worse prognosis than pCA IX alone (*P*=0.05). A model was then developed where the study population was divided into three groups: group 1 had expression of EGFR without coexpression of MMP-9 or pCA IX (number=21); group 2 had no expression of EGFR (number=75); and group 3 had coexpression of EGFR with pCA IX or MMP-9 or both (number=70). Group 3 had a worse prognosis than either groups 1 or 2 (*P*=0.0003 and 0.027, respectively) and group 1 had a better prognosis than group 2 (*P*=0.036). These data identify two cohorts of EGFR-positive patients with diametrically opposite prognoses. The group expressing either EGFR and or both MMP-9 and pCA IX may identify a group of patients with activated EGFR, which is of clinical relevance with the advent of EGFR-targeted therapies.

Epidermal growth factor receptor (EGFR) is a member of the c-erbB membrane receptor family and was first described in 1980 (Cohen *et al*, 1980). Epidermal growth factor receptor signalling promotes angiogenesis, cell proliferation, tumour invasion and inhibits tumour suppressor gene activity and apoptotic signalling (Westermark *et al*, 1982; Abdollahi *et al*, 1999; Rosen *et al*, 2001; Gildea *et al*, 2002; Hirata *et al*, 2002; Di Gennaro *et al*, 2003). Recently, the clinical relevance of the EGFR has been heightened in light of the development of the EGFR tyrosine kinase inhibitors, Getifinib and Erlotinib and EGFR monoclonal antibodies Cetuximab that have been demonstrated to have antitumour activity in solid tumours including non-small cell lung cancer (NSCLC) (Giaccone *et al*, 2004; Herbst *et al*, 2004a, 2004b; Lynch *et al*, 2004b).

Immunohistochemical studies have reported EGFR overexpression in 22–81% of NSCLC tumours depending on the antibody used and cut point that defines overexpression (Veale *et al*, 1987; Volm *et al*, 1993; Pfeiffer *et al*, 1996; Cornianu and Tudose, 1997; Pastorino *et al*, 1997; Rusch *et al*, 1997; Fontanini *et al*, 1998; Greatens *et al*, 1998; D'Amico *et al*, 1999; Fu *et al*, 1999; Cox *et al*, 2000; Ohsaki *et al*, 2000; Selvaggi *et al*, 2002; Hirsch *et al*, 2003; Kanematsu *et al*, 2003; Mukohara *et al*, 2003; Onn *et al*, 2003). The majority of these studies have failed to demonstrate an association with prognosis (Table 1

**Table 1:** Immunohistochemical studies of EGFR expression in NSCLC

**No.**	**Staining pattern**	**Stage**	**Cut point**	**Percentage overexpressed**	***P*-value**	**EGFR antibody**	**Refs.**
169	m and c	I–IIIA	⩾20%	56%	0.17	Novacastra Labs EGFR.113	Cox *et al* (2000)
96		I–IIIA	⩾20%	32%	N/S		Rusch *et al* (1997)
186	M	I–IV	0%, 0<80%, ⩾80%	14, 31, 55%	0.9	R1, Amersham	Pfeiffer *et al* (1996)
515		I	<10%	50, 47%	0.25	31G7, Triton	Pastorino *et al* (1997)
290		I–IV		43%	0.02*υ*		Ohsaki *et al* (2000)
158		I–IIIA	⩾0%	66%	0.36	Sigma 014H4819	Fu *et al* (1999)
77	m and c	I–IV	0−+++		<0.05*κ*	EGFR1	Veale *et al* (1987)
121					N/S		Volm *et al* (1993)
195		I–IIIA	⩾45%	53%/47%	0.80	Anti-EGFR Triton	Fontanini *et al* (1998)
408		I			N/S		D'Amico *et al* (1999)
183	M	I–III	Tertiles	38%/25%/37%	0.22	Zymed Labs No. 28–0005	Hirsch *et al* (2003)
					υ		Cornianu and Tudose (1997)
101		I–III	++/+++ intensity ⩾5%	35%/66%	N/S	Zymed Labs clone 31G7	Greatens *et al* (1998)
60		I–III	⩾30%	22%/78%	0.6	Zymed Labs clone 31G7	Mukohara *et al* (2003)
36		I–III		81%/19%	N/S		Kanematsu *et al* (2003)
98	M	I	⩾10%	61%/39%	N/S		Onn *et al* (2003)
130		I–III	⩾10%	37%/63%	<0.01*υ*	Oncogene Ab-1	Selvaggi *et al* (2002)

EGFR=epidermal growth factor receptor; S=significant; N/S=not significant; *υ* associated with a poor prognosis; *κ* associated with stage survival analysis not performed.

). This may be because EGFR activation usually requires the binding of specific ligands prior to induction of phosphorylation of the tyrosine domain of the receptor and subsequent dimerisation with either another EGFR molecule or with another member of the c-erbB family of receptors (Cohen *et al*, 1981; Yarden and Ullrich, 1988; Tzahar *et al*, 1996). Hence, expression of EGFR alone may not accurately represent EGFR activity. However, coexpression of EGFR with neither its most common dimerisation partner, C-erbB2, nor its most common ligand, transforming growth factor (TGF) *α*, predict prognosis (Rusch *et al*, 1997; Kanematsu *et al*, 2003). Despite these observations, studies that are able to identify patients with activated EGFR may identify patients with a poor outcome. This contention is supported by a small study of 36 patients with resected NSCLC tumours, which reported that phosphorylated EGFR expression is associated with a poor prognosis (Kanematsu *et al*, 2003). Therefore, coexpression of EGFR with downstream factors may identify such patients and add credence to this hypothesis.

In our series of patients with NSCLC, the expression of EGFR was not associated with prognosis (Cox *et al*, 2000). However, an important relationship was found between EGFR and matrix metalloproteinase (MMP)-9 expression. Matrix metalloproteinase-9 is an enzyme involved in the degradation of the extracellular matrix and increased expression was associated with a poor prognosis. Coexpression of EGFR and MMP-9 identified a subset of patients with a significantly worse prognosis than either EGFR or MMP-9 alone (Cox *et al*, 2000). *In vitro* experiments demonstrate that EGF stimulation of EGFR-positive NSCLC cell lines can result in the upregulation of MMP-9 and, likewise, inhibition of EGFR *in vivo* reduces tumour cell MMP-9 expression (Perrotte *et al*, 1999; O'Byrne *et al*, 2001). These data suggest that coexpression of MMP-9 and EGFR may identify patients with activated EGFR.

Carbonic anhydrase (CA) IX is a marker of hypoxia and is regulated by the transcription factor hypoxia-inducible factor (HIF)-1*α* (Wykoff *et al*, 2000; Swinson *et al*, 2003). Expression of CA IX is associated with a poor prognosis in NSCLC (Giatromanolaki *et al*, 2001; Swinson *et al*, 2003). EGF treatment of tumour cell lines induces HIF-1*α* expression and constitutively active mutations of EGFR potentiate hypoxic induction of other targets of HIF-1*α* such as VEGF (Clarke *et al*, 2001; Semenza, 2002). Giatromanolaki *et al* reported an association between CA IX and EGFR and we have reported an association between EGFR and HIF-1*α* (Giatromanolaki *et al*, 2001; Swinson *et al*, 2004). These data suggest that coexpression of CA IX and EGFR may also identify patients with activated EGFR.

The aims of this study were to first update the survival data from previous studies of EGFR, MMP-9 and CA IX in a series of surgically resected NSCLC, assess if an association exists between EGFR and CA IX and develop a model using EGFR and related downstream factor expression to predict the outcome in NSCLC.

## MATERIALS AND METHODS

### Ethics

The Leicester locoregional ethical committee granted ethical approval for these studies.

### Patient inclusion and exclusion criteria and follow-up

A consecutive series of patients who had had NSCLC tumours resected with curative intent were considered for entry into the two studies. Patients were excluded if tissue from the resected specimen was not available, if they had pathologically staged stage IV disease or survival of less than 61 days from time of operation so as to exclude the confounding factor of perioperative mortality (Cox *et al*, 2000; Swinson *et al*, 2003). The final staging was based on the findings at surgery and the histopathology report. Hospital notes of the patients were reviewed, and if necessary, the local cancer registries or patient's general practitioner were contacted to complete case follow-up.

### Immunohistochemistry

The specimens had previously been evaluated for the expression of EGFR, MMP-9 and CA IX. Standard immunohistochemical methods were employed using the anti-EGFR mouse monoclonal antibody (Mab) EGFR.113 (Novocastra Laboratories Ltd, Newcastle, UK) (Cox *et al*, 2000), anti-MMP-9 mouse Mab 56-2A4 (Chemicon International Ltd, Temecula, CA 92590, USA) (Cox *et al*, 2000) and anti-CA IX antibody M75 (Gift from Professor J Pastorek, Institute of Virology, Slovak Academy of Sciences, Slovak Republic) (Table 2

**Table 2:** Immunohistochemistry techniques and antibodies

	**EGFR**	**MMP-9**	**CA IX**
Antigen retrieval	Pressure cooking 2 min	Pressure cooking 2 min	No antigen retrieval
Blocking serum	Rabbit	Rabbit	Human
Primary antibody	Novacastra EGFR.113	Chemicon 56-2A6	M75
Dilution	1 : 20	1 : 100	1 : 50
Incubation	Overnight 4°C	Overnight 4°C	30 min at 20°C
Secondary antibody	Rabbit anti-mouse	Rabbit anti-mouse	Goat anti-mouse Ig
	Ig (Dako)	Ig (Dako)	(Envision Kit, Dako)
Dilution	1 : 400	1 : 400	Neat
Buffer	100 mmol Tris, 300 mmol NaCl TBS, pH 7.65	100 mmol Tris, 300 mmol NaCl TBS, pH 7.65	100 mmol Tris, 300 mmol NaCl TBS, pH 7.65
IHC kit	ABC (Dako)	ABC (Dako)	Envision (Dako)
Cut point for categorical analysis	⩾20%	⩾20%	⩾5% (mCA IX)
			>0% (pCA IX)
Reference	Cox *et al* (2000)	Cox *et al* (2000)	Swinson *et al* (2003)

EGFR=epidermal growth factor receptor; MMP-9=metalloproteinase-9; pCA=perinuclear carbonic anhydrase; mCA=membranous carbonic anhydrase.

) (Swinson *et al*, 2003).

### Interpretation

The percentage of cells staining positively in each study was estimated using light microscopy. The cut points used to dichotomise the series in each study were predetermined. In all, 20% plus tumour cell staining was used as a cut point to define overexpression of EGFR (both cytoplasmic and membranous) and MMP-9 (cytoplasmic) (Cox *et al*, 2000). Two independent investigators, blinded from the other's results interpreted the slides and where a discrepancy was found a consensus was reached using a double-headed microscope. In the second study, there were three distinct patterns of CA IX staining perinuclear (p), membranous (m) and cytoplasmic. The presence or absence of pCA IX staining was used as a cut point, as this pattern of staining was an infrequent observation. Greater or equal to the median defined high mCA IX staining (Swinson *et al*, 2003). Two investigators blinded from each other's results again interpreted the staining. A third investigator adjudicated the result where discrepancies were found. The survival data from these studies were reviewed and updated.

### Literature search for studies investigating EGFR expression in NSCLC

Pubmed, Embase, Medline databases, the Cochrane library and ASCO annual meeting abstracts were searched using EGFR, NSCLC and immunohistochemistry as key words.

### Statistical analysis

The SPSS software system (SPSS for Windows Version 9.0) was used to perform the statistical analysis. The *χ*^2^-test was used to analyse the associations between categorical variables. A *P*-value of ⩽0.05 was used as the level of significance. Overall survival as opposed to cancer specific mortality was used to avoid bias. Survival curves were plotted using the Kaplan–Meier method and a log-rank test was used to assess the statistical significance of differences in survival. A Cox proportional-hazards regression model was used to investigate whether coexpression of factors with EGFR significantly worsened outcome compared to expression of factors in isolation. A Cox proportional-hazards regression model was also used to identify statistically significant differences in survival and estimate hazard ratios and 95% confidence intervals (CI). Covariables were entered into the model if *P*⩽0.05 and removed if *P*⩾0.1.

## RESULTS

### Study population

In all, 218 patients were considered for the two studies. Of these, 24 patients were excluded due to poor postoperative survival and 13 patients were excluded as they were found to have pathological stage IV disease. Of the 181 patients, tissue was available from 179 patients for staining for EGFR expression, from 177 patients for staining for CA IX expression and from 169 patients for staining for MMP-9 expression. In total, there were 166 cases stained for all three markers. Of the 166 patients available for analysis, 115 (69.3%) were male and 51 (30.7%) were female. A total of 82 (49.4%) patients had stage I, 46 (27.7%) patients had stage II and 38 patients had stage IIIA (22.9%) disease. A total of 47 (28.2%) patients had adenocarcinoma, 101 (60.8%%) patients had squamous carcinoma, 14 (8.4%) patients had large-cell carcinoma and four (2.4%) patients had tumours that were not characterised. The mean age at surgery was 65 years (s.d. 7.9, range 33.8–79.1). Positive resection margins were found in 15 patients. One patient had received adjuvant chemotherapy. Adjuvant radiotherapy was given to 17 patients, of whom 10 were stage IIIA, six were stage II and one was stage I.

In total, 126 (75.9%) patients had died at the time of analysis and of these 18 (10.8%) were not cancer related. The duration of follow-up from the time of surgery was between 5 and 10 years.

Of the 15 patients who had been excluded due to lack of tissue, there was no statistical difference in the stage (*P*=0.72), histology (*P*=0.9), sex (*P*=0.56) or adjuvant radiotherapy (*P*=0.56) distribution compared to the patients used in the survival analysis.

### Associations between different patterns of CA IX staining and EGFR

Using the *χ*^2^ test membranous carbonic anhydrase (mCA) IX and pCA IX expression patterns were positively associated with EGFR expression. The association between pCA IX and EGFR expression was the strongest (*P*<0.001). All the pCA IX-positive tumours expressed mCA IX. The association between the mCA IX group and EGFR was dependent on the pCA IX-positive cases, as it was lost when the pCA IX group was subtracted from the series (*P*=0.93) (Table 3

**Table 3:** Frequency table for pCA IX, EGFR and MMP-9 expression

**Factor**	**Negative pCA IX**	**Positive pCA IX**	***χ*^2^ *P*-value**
*EGFR (N*=*176)*			
<20%	73	11	<0.001
⩾20%	58	34	
			
*MMP-9 (N*=*166)*			
Low	61	18	0.09
High	59	28	
			
	**Low mCA IX**	**High mCA IX**	
*EGFR (N*=*176)*			
<20%	53	31	0.009
⩾20%	40	52	
			
	**Low mCA IX**	**High mCA IX**	
*pCA IX-positive cases subtracted EGFR (N*=*130)*			
<20%	50	22	0.79
⩾20%	39	19	

EGFR=epidermal growth factor receptor; MMP-9=metalloproteinase-9; pCA=perinuclear carbonic anhydrase; mCA=membranous carbonic anhydrase.

). Perinuclear CA IX was therefore used in survival analyses for this review.

There was a trend for a positive association between pCA IX and MMP-9 expression (*P*=0.09) (Table 3).

### Survival analysis for EGFR-related variables

**Figure 1:**
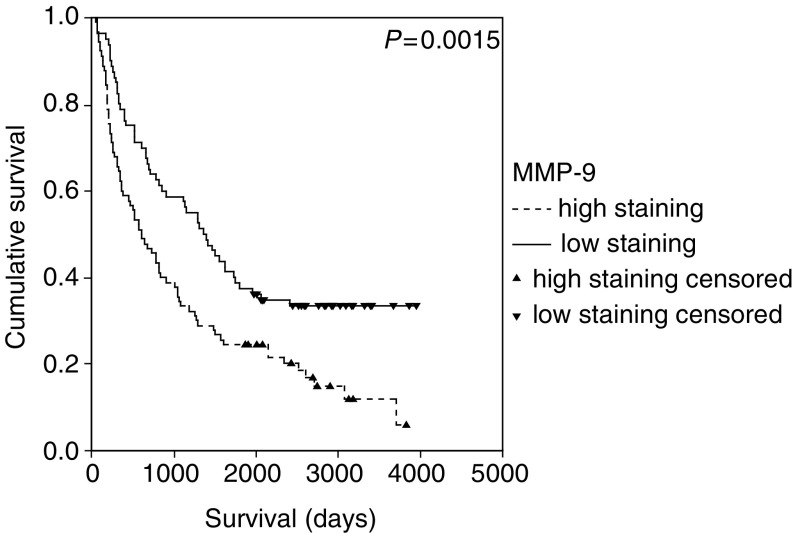
Kaplan–Meier survival curve and log-rank *P*-value for MMP-9 expression in NSCLC.

Survival data for EGFR, pCA IX and MMP-9 expression were updated for the study and there was no significant change in the previously reported outcomes (data not shown) (Cox *et al*, 2000; Swinson *et al*, 2003). Using the log-rank test, MMP-9 (*P*=0.0015) and pCA IX (*P*=0.03) were associated with a poor prognosis and EGFR expression had no prognostic value (*P*=0.72) (Figures 1

, 2

**Figure 2:**
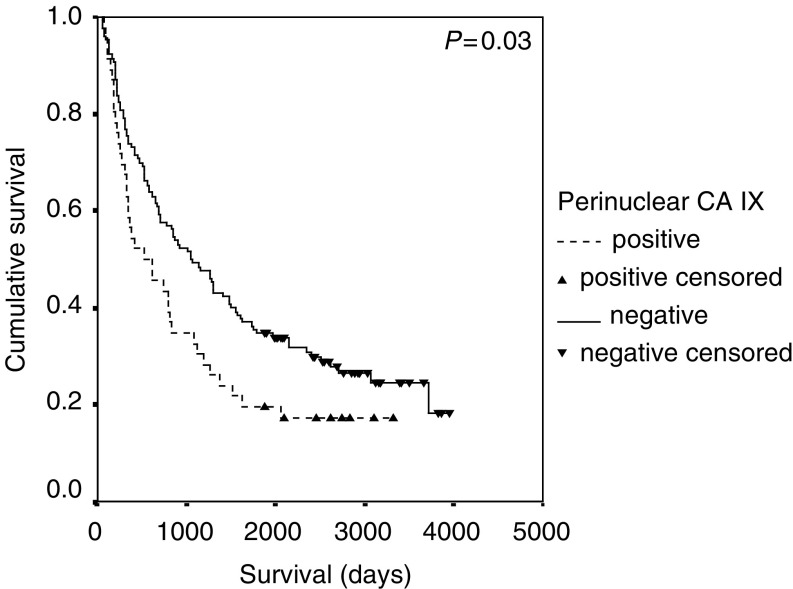
Kaplan–Meier survival curve and log-rank *P*-value for pCA IX expression in NSCLC.

and 3

**Figure 3:**
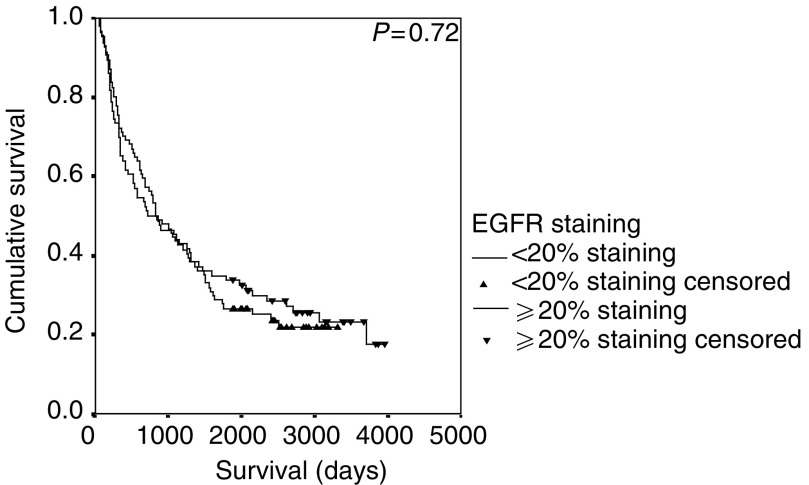
Kaplan–Meier survival curve and log-rank *P*-value for EGFR expression in NSCLC.

).

Using Cox's regression analysis to estimate hazard ratios, coexpression of EGFR with pCA IX, mCA IX or MMP-9 increased the hazard ratio value and strengthened the *P*-value compared to these variables alone (Table 4

**Table 4:** Univariate survival of EGFR-related biological variables using Cox's regression analysis

**Prognostic factor**	***N***	**Hazard ratio**	**95% CI**	***P*-value**
*EGFR*				
<20%	86	1		0.64
⩾20%	93	1.08	0.77–1.51	
		179		
*MMP-9*				
<20%	80	1		0.001
⩾20%	89	1.79	1.26–2.55	
		169		
				
*EGFR/MMP-9 coexpression*				
−ve	61	1.00		<0.0001
+ve	107	2.19	1.54–3.1408	
		168		
*pCA IX*				
Positive	46	1.0		0.044
Negative	131	1.50	1.03–2.19	
	177			
				
*EGFR/pCA IX coexpression*				
−ve	142	1		0.003
+ve	34	1.86	1.24–2.80	
	176			

EGFR=epidermal growth factor receptor; MMP-9=metalloproteinase-9; pCA=perinuclear carbonic anhydrase; 95% CI=95% confidence intervals.

). By entering these variables into a multivariate analysis model, the increase in the hazard ratio for pCA IX and MMP-9 when coexpressed with EGFR was shown to be significant (Tables 5

**Table 5:** Cox's regression model for EGFR/MMP-9 coexpression

**Variable**	**Harzard ratio**	**95% CI**	***P*-value**
*MMP-9*			
<20%	1.0		0.792
⩾20%	0.93	0.54–1.59	
			
*EGFR*			
<20%	1.0		0.015
⩾20%	0.52	0.31–0.88	
			
*MMP-9/EGFR coexpression*			
Negative	1.0		<0.001
Positive	3.55	1.73–7.26	

EGFR=epidermal growth factor receptor; MMP-9=metalloproteinase-9; 95% CI=95% confidence intervals.

and 6

**Table 6:** Cox's regression model for EGFR/pCA IX coexpression

**Variable**	**Hazard ratio**	**95% CI**	***P*-value**
*pCA IX*			
Negative	1.0		0.781
Positive	0.90	0.42–1.89	
			
*EGFR*			
<20%	1.0		0.172
⩾20%	0.75	0.50–1.13	
			
*pCA IX/EGFR coexpression*			
Negative	1.0		0.05
Positive	3.55	1.0–5.85	

EGFR=epidermal growth factor receptor; pCA=perinuclear carbonic anhydrase; 95% CI=95% confidence intervals.

). However, this was not the case for coexpression of mCA IX.

### Survival analysis for EGFR coexpression, no coexpression and no EGFR expression

**Figure 4:**
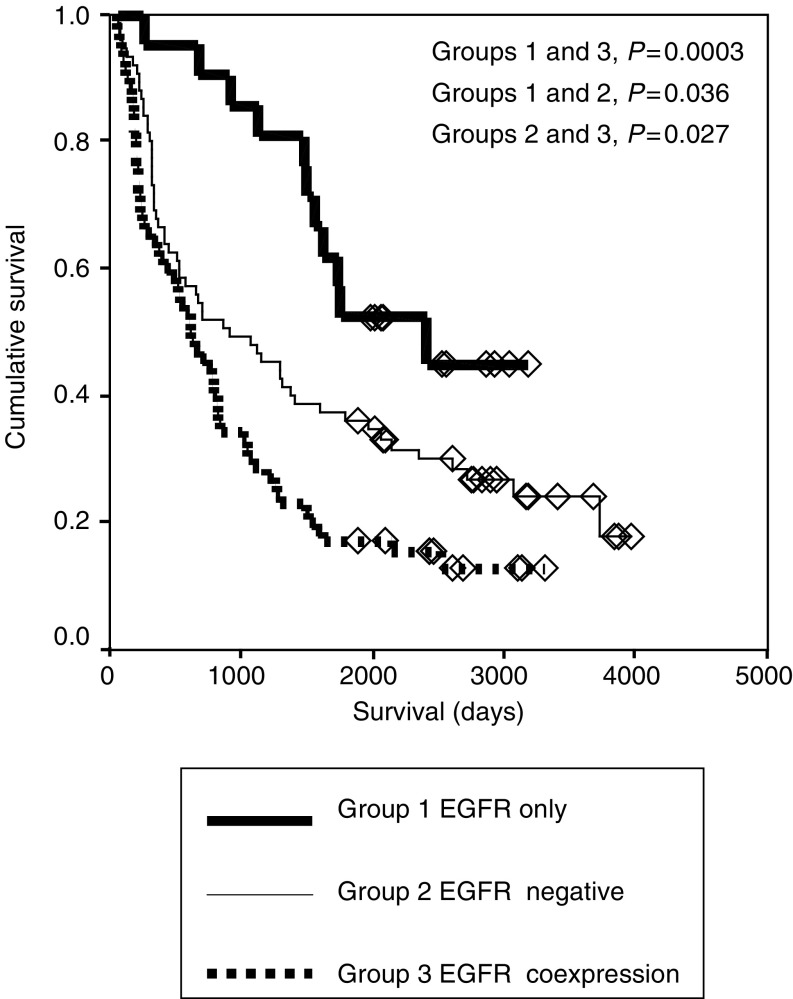
Survival curves for EGFR expression alone (group 1), EGFR negative (group 2) and coexpression of EGFR with either MMP-9 or CA IX or both (group 3).

In view of the associations between pCA IX, MMP 9 and EGFR expression, the study population was divided into three groups: group 1 expression of EGFR in the absence of pCA IX and MMP-9; group 2 no expression of EGFR; and group 3 coexpression of EGFR with either pCA IX or MMP-9 or both. Using the log-rank test, group 3 had a worse prognosis than either groups 1 or 2 (*P*=0.0003 and 0.027, respectively) and group 1 had a better prognosis than group 2 (*P*=0.036) (Figure 4

) (Cox's regression analysis, Table 7

**Table 7:** Cox's regression analysis for pCA IX, MMP-9 and EGFR coexpression

**Hazard ratio**	**95% CI**	***P*-value**
*No EGFR expression (N*=*21)*		
1.0		0.0002
		
*EGFR only (N*=*75)*		
0.49	0.93–0.26	0.031
		
*EGFR/MMP-9 or pCA IX coexpression (N*=*70)*		
3.25	1.70–6.20	0.0004

EGFR=epidermal growth factor receptor; MMP-9=metalloproteinase-9; pCA=perinuclear carbonic anhydrase; 95% CI=95% confidence intervals.

). Using the *χ*^2^ test, the pattern of EGFR expression (membranous, cytoplasmic or mixed) did not differ between the groups.

### Multivariate analysis

The clinicopathological factors, stage, gender, positive margins and the use of adjuvant radiotherapy, were entered into a multivariate model with the three groups. Stage, gender and Epidermal growth factor receptor groupings were independent prognostic variables (Table 8

**Table 8:** Multivariate analysis of clinicopathological variables and EGFR groupings

**Number**	**Variable**	**Hazard ratio**	**95% CI**	***P*-value**
82	Stage 1	1.0		0.001
46	Stage 2	1.55	1.02–2.37	0.04
38	Stage 3A	2.26	1.47–3.49	0.0002
				
51	Female	1.0		
115	Male	1.76	1.17–2.66	0.007
				
21	EGFR only	1.0		0.0003
75	No EGFR	2.11	1.1–4.08	0.025
70	EGFR coexpression	3.26	1.69–6.27	0.0004

EGFR=epidermal growth factor receptor; 95% CI=95% confidence intervals.

).

## DISCUSSION

EGFR expression was closely associated with CA IX expression in agreement with the study by Giatromanolaki *et al* We have previously reported two important patterns of CA IX staining in NSCLC, mCA IX, which has been proposed to be a marker of tumour cell hypoxia and pCA IX that is associated with a poor prognosis (Swinson *et al*, 2003). pCA IX was closely related to the mCA IX group as all pCA IX tumour cells expressed mCA IX and the majority of pCA IX positive cases (42 of 46) had high mCA IX expression. The association between EGFR and pCA IX was stronger than between mCA IX and EGFR. The latter relationship appeared to be dependent on the former as when the pCA IX subgroup was subtracted from the series the relationship between mCA IX and EGFR was lost. When EGFR was coexpressed with pCA IX, a worse prognosis was observed than when either of these factors were expressed on their own, hence mirroring the relationship between MMP-9 and EGFR (Cox *et al*, 2000). As such, a model was developed where the series was split into three groups. The first group expressed EGFR alone; the second did not express EGFR and the third expressed EGFR with either MMP-9 or pCA IX or both. The prognosis of the third group was the worst, whereas the prognosis of the first was the best.

The polarisation of prognosis depending on whether or not EGFR is coexpressed with related factors provides grounds for the hypothesis that coexpression of EGFR with either MMP-9 or pCA IX or both represents patients with activated EGFR. This hypothesis explains the differences in prognosis between group 3 and the other two groups. This hypothesis is also supported by a small study that has reported that phosphorylated EGFR is associated with a poor prognosis in NSCLC (Kanematsu *et al*, 2003).

The difference between groups 1 and 2 is less easily explained. One explanation could be that some patients in group 2 expressed pCA IX and MMP-9, both markers of a poor prognosis, whereas by definition no patients in group 1 expressed these factors. However, subtraction of patients with either MMP-9 or pCA IX or both expression from group 2 did not alter the survival difference between the two groups (data not shown).

Alternatively in the downstream marker negative patients, EGFR may stimulate proapoptotic pathways. Recent work has shown that tumour cell lines expressing high levels of EGFR may undergo apoptosis, particularly following exposure to EGF. Increasing the level of EGFR expression in a variety of cell types predictably leads to apoptosis, a process that requires an active tyrosine kinase but not EGFR autophosphorylation sites (Gulli *et al*, 1996; Hognason *et al*, 2001). Further clinical evidence for a beneficial effect has been observed in patients receiving cisplatin chemotherapy for advanced NSCLC, where EGFR expression has been associated with a better prognosis (Bailey *et al*, 2004).

In summary, we have demonstrated an association between EGFR and different patterns of CA IX expression and have previously demonstrated a similar relationship between EGFR and MMP-9. We have hypothesised that cases with coexpression of EGFR with either MMP-9 or pCA IX or both represent cases with activated EGFR. Hence, promoting an aggressive NSCLC phenotype. By developing an assay to select cases with activated EGFR, a cohort of patients may be identified that are highly responsive to anti-EGFR therapy. Such studies are under way using specimens collected from the large number of clinical trials exploring EGFR-targeted therapies in NSCLC (Lynch *et al*, 2004a; Paez *et al*, 2004). The implications of such a finding may have great clinical benefits.
